# Outcomes After Transcatheter Aortic Valve Implantation Using the Self-Expanding Allegra Valve in Daily Practice

**DOI:** 10.14740/cr2291

**Published:** 2026-10-24

**Authors:** Justina Zemaite, Stefan Erler, Ulrich Schafer, Steffen Vormfelde, Bjoern Andrew Remppis, Katja Bohmann, Barbara Maria Januszewski, Christof Burgdorf

**Affiliations:** a Department of Cardiology, Heart and Vascular Center Bad Bevensen, Bad Bevensen, Germany; b Department of Cardiothoracic Surgery, Heart and Vascular Center Bad Bevensen, Bad Bevensen, Germany; c Department of Cardiac Surgery, Helios Clinic Siegburg, Siegburg, Germany

**Keywords:** Transcatheter aortic valve implantation, Allegra, Aortic stenosis, Valve-in-valve, Outcome

## Abstract

**Background:**

Limited data are available of the self-expanding Allegra transcatheter heart valve (THV). The aim of this study was to investigate the safety and outcome of transcatheter aortic valve implantation (TAVI) using the Allegra THV in daily practice.

**Methods:**

Between February 2018 and September 2024, 142 consecutive patients underwent Allegra TAVI at the Heart and Vascular Center, Bad Bevensen, Germany, either for native aortic stenosis (AS) (n = 117, median 82 (interquartile range 80–86) years) or degenerated aortic bioprostheses (n = 25, 80 (73–84) years) as valve-in-valve (ViV) procedures. Endpoints were defined according to Valve Academic Research Consortium-3 criteria.

**Results:**

The logistic EuroSCORE II was significantly higher in the cohort of patients with degenerated aortic bioprostheses than in those with native AS (14.4% (6.6–25.3%) versus 3.3% (2.5–5.7%), P < 0.01). Technical success was nearly identical in both cohorts (91.5% of patients with native AS and 92.0% of patients with ViV TAVI). Postinterventional aortic regurgitation ≥ moderate was found in 4.5% of patients with native AS and was not present in any patient with ViV TAVI. In the native AS cohort, mean and maximal aortic pressure gradients decreased from 44 (36–54) mm Hg and 72 (60–87) mm Hg before the intervention to 6 (5–9) mm Hg and 12 (9–15) mm Hg after Allegra TAVI. In the ViV cohort, mean and maximal transvalvular pressure gradients decreased from 34 (27–43) mm Hg and 59 (44–70) mm Hg to 9 (7–11) mm Hg and 16 (14–23) mm Hg, respectively. Periprocedural mortality rates (≤ 30 days) were 6.8% and 12.0% in the native AS and ViV cohorts, respectively. One-year and 3-year survival rates were 87.2% and 73.2% in the native AS cohort and 89.8% and 75.1% in the ViV cohort, respectively.

**Conclusions:**

TAVI using the self-expanding Allegra THV system is safe and leads to acute hemodynamic improvements in patients with native AS or degenerated aortic bioprostheses.

## Introduction

The Allegra transcatheter heart valve (THV) system (New Valve Technology/Biosensors, Hechingen, Germany) consists of a self-expanding supra-annular bovine pericardial aortic bioprosthesis and a transvascular delivery system that, in contrast to other self-expanding systems, is characterized by the absence of an occlusive phase during valve implantation. Although the Allegra THV system already received Conformité Européenne approval in March 2017 for the treatment of patients with native aortic stenosis (AS) and in April 2020 for the treatment of patients with failing surgical aortic bioprostheses as valve-in-valve (ViV) procedure, and there have been no significant changes since then, it is noteworthy that only a very limited amount of data is available for the Allegra system.

Short-term results according to the Valve Academic Research Consortium (VARC)-2 criteria after Allegra transcatheter aortic valve implantation (TAVI) indicate an acute device success rate ranging from 85.7% to 95.7% in patients with native AS and 96.6% in patients with degenerated aortic bioprostheses [1–5]. Only recently, the use of the Allegra THV has been suggested as an alternative to the established THV, such as CoreValve/Evolut R and Sapien for ViV TAVI, especially in patients with previously implanted small aortic prostheses [6].

Therefore, this single-center study sought to evaluate “real world” periprocedural data and long-term outcomes after TAVI using the self-expanding Allegra valve in daily routine.

## Materials and Methods

This retrospective observational study complied with the guidelines for human studies and was conducted in accordance with the World Medical Association Declaration of Helsinki. The study protocol was approved by the Institutional Review and Safety Board of the Heart and Vascular Center Bad Bevensen.

### Patients

Between February 2018 and September 2024, 1,993 consecutive patients underwent TAVI at our institution. Of these, 156 patients were intended to treat using the Allegra THV. Fourteen patients were excluded from the present analysis because of their participation in a parallel study using the Allegra THV [7]. All patients were evaluated by an interdisciplinary heart team and were eligible for TAVI due to increased surgical risk for (re)operation. All patients provided written informed consent prior to implantation.

### Device

The Allegra THV consists of bovine pericardial leaflets attached to a nitinol stent frame in a supra-annular position (Fig. 1). The frame design allows for a variable radial force along the stent, with safe anchoring of the prosthesis within the native aortic annulus or prior implanted aortic bioprostheses. To reduce stress on the leaflets, the upper stent frame allows pole movement, and the ventricular inner inflow part is covered by a bovine sealing skirt to prevent paravalvular leakage (Fig. 1).

**Figure 1:**
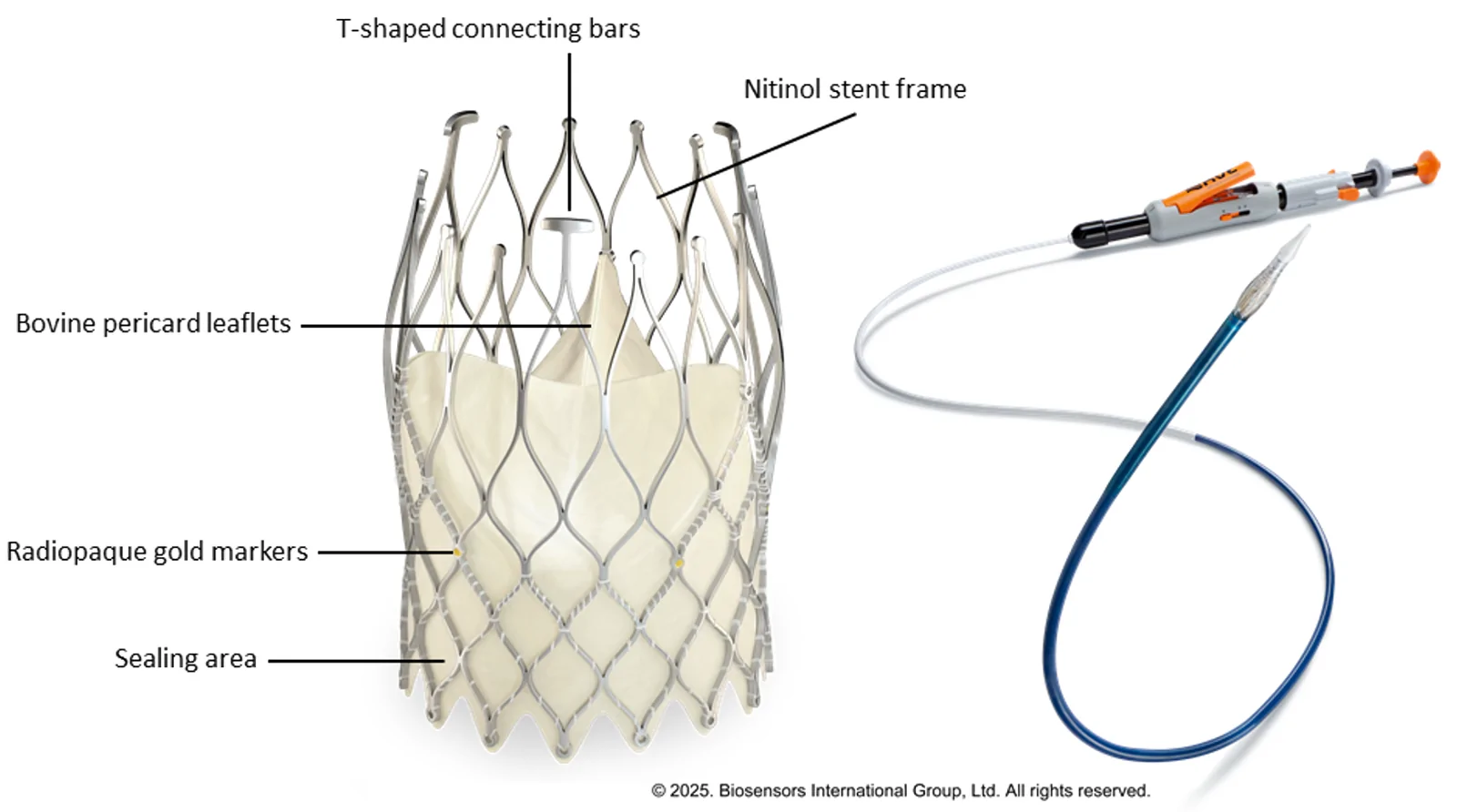
Allegra transcatheter heart valve and transvascular delivery system.

### Procedure

TAVI was performed according to standard techniques with transfemoral vascular access in all but two cases, which were performed via a subclavian access route. For the choice of the specific Allegra THV size, the aortic root dimensions (as determined by multislice computed tomography, angiography, and echocardiography) as well as the size of the existing aortic bioprosthesis were considered. To define the perpendicular annulus plane by fluoroscopy and to obtain the alignment of the right cusp between the left and the non-coronary cusp, root angiography was performed with the pigtail catheter placed in the right coronary sinus [8]. In patients with degenerated aortic bioprosthesis, the Allegra THV was aligned primarily to the stent frame of the previously implanted bioprosthesis. Fast (about 120 beats per minute (bpm)) or rapid (about 180 bpm) ventricular pacing was applied to suppress premature ventricular contractions and cardiac stroke volume in conjunction with aortic route angiography to confirm the final device alignment during deployment. All TAVI procedures were performed in a hybrid operating room under general anesthesia.

Patient and technical data were assessed before and 30 days after intervention. Mid- and long-term survival were assessed 1 and 3 years after the procedure. Transthoracic and transesophageal echocardiographic data were assessed using standard techniques. Endpoints were defined according to the VARC-3 criteria [9].

### Statistical analysis

Continuous data are presented as median and interquartile range, and frequencies and percentages are used to describe discrete variables. Continuous data were analyzed using the Wilcoxon matched-pairs signed rank test and unpaired Mann–Whitney nonparametric U test, and categorical variables were analyzed using two-tailed Fisher’s exact test. Survival curves were compared using the log-rank (Mantel-Cox) test. A P value less than 0.05 was considered statistically significant. Statistical analyses were performed using GraphPad Prism 7.0 (GraphPad Software Inc., San Diego, CA, USA).

## Results

### Clinical characteristics

The baseline clinical characteristics of all the patients treated with Allegra THV are summarized in Table 1. The median age was 82 (80–85) years and 64.8% of the patients were women. Logistic EuroSCORE I (17.4% (12.1–26.07%)) and II (4.0% (2.6–7.3%)) fit an intermediate risk profile (Table 1). Severe native AS was present in 117 of the 142 patients (82.4%). The remaining 25 patients (17.6%) underwent ViV procedures because of degenerated aortic bioprostheses. The clinical characteristics of both cohorts are shown in Table 2. Significant differences at baseline were found with respect to age, New York Heart Association functional class, prior coronary artery bypass grafting, extracardiac arteriopathy, bundle branch block on electrocardiogram, prior pacemaker/implantable cardioverter defibrillator implantation, left ventricular ejection fraction, left ventricular end-diastolic volume, concomitant mitral and tricuspid regurgitation, renal function, and N-terminal pro-brain natriuretic peptide (NT-proBNP) levels. Accordingly, the logistic EuroSCORE I and II were significantly higher in patients who underwent ViV TAVI.

**Table 1:** Clinical Characteristics of All Patients (N = 142) at Baseline

Age (years)	82 (80–85)
Female/male (n)	92/50
Body mass index (kg/m^2^)	27 (24–31)
NYHA classification ≥ III (n)	77 (54.2%)
Coronary artery disease (n)	96 (67.6%)
Prior MI (n)	15 (10.6%)
Prior PCI (n)	36 (25.4%)
Prior CABG (n)	20 (14.1%)
COPD (n)	15 (10.6%)
Extracardiac arteriopathy (n)	34 (23.9%)
Previous stroke/TIA (n)	15 (10.6%)
ECG	
Sinus rhythm (n)	91 (64.1%)
AV block (n)	25 (17.6%)
Bundle branch block (n)	35 (24.6%)
Atrial fibrillation (n)	64 (45.1%)
Pacemaker/ICD (n)	22 (15.5%)
TTE/TEE	
LVEF (%)	57 (50–63)
LVEDV (mL)	86 (68–109)
Mitral regurgitation ≥ II (n)	35 (24.6%)
Tricuspid regurgitation ≥ II (n)	22 (15.5%)
Systolic PAP (mm Hg)	38 (31–46)
Creatinine (mg/dL)	1.0 (0.7–1.2)
GFR (mL/min)	63 (46–80)
NT-proBNP (ng/L)	1,713 (659–5,212)
Logistic ES I (%)	17.4 (12.1–26.7)
Logistic ES II (%)	4.0 (2.6–7.3)

Data are presented as median, interquartile range, frequency, and percentage. AV: atrioventricular; CABG: coronary artery bypass grafting; COPD: chronic obstructive pulmonary disease; ECG: electrocardiogram; ES: EuroSCORE; GFR: glomerular filtration rate; ICD: implantable cardioverter defibrillator; LVEF: left ventricular ejection fraction; LVEDV: left ventricular end-diastolic volume; MI: myocardial infarction; NT-proBNP: N-terminal pro-brain natriuretic peptide; NYHA: New York Heart Association; PAP: pulmonary artery pressure; PCI: percutaneous coronary intervention**;** TIA: transient ischemic attack; TEE: transesophageal echocardiography; TTE: transthoracic echocardiography.

**Table 2:** Clinical Characteristics at Baseline Separated for Patients With Native Aortic Stenosis (AS) or Degenerated Aortic Bioprosthesis and Valve-in-Valve (ViV) Procedure

	Native AS (n = 117)	ViV (n = 25)
Age (years)	82 (80–86)	80 (73–84)**
Female/male (n)	79/38	13/12
Body mass index (kg/m^2^)	28 (24–31)	26 (23–29)
NYHA classification ≥ III (n)	58 (49.6%)	19 (76.0%)*
Coronary artery disease (n)	77 (65.8%)	19 (76.0%)
Prior MI (n)	12 (10.3%)	3 (12.0%)
Prior PCI (n)	29 (24.8%)	7 (28.0%)
Prior CABG (n)	9 (7.7%)	11 (44.0%)**
COPD (n)	12 (10.3%)	3 (12.0%)
Extracardiac arteriopathy (n)	20 (17.1%)	14 (56.0%)**
Previous stroke/TIA (n)	13 (11.1%)	2 (8.0%)
ECG		
Sinus rhythm (n)	76 (65.0%)	15 (60.0%)
AV block (n)	18 (15.4%)	7 (28.0%)
Bundle branch block (n)	24 (20.5%)	11 (44.0%)*
Atrial fibrillation (n)	52 (44.4%)	12 (48.0%)
Pacemaker/ICD (n)	13 (11.1%)	9 (36.0%)**
TTE/TEE		
LVEF (%)	57 (53–63)	49 (34–59)**
LVEDV (mL)	81 (65–100)	119 (81–178)**
Mitral regurgitation ≥ II (n)	22 (18.8%)	13 (52.0%)**
Tricuspid regurgitation ≥ II (n)	14 (12.0%)	8 (32.0%)*
Systolic PAP (mm Hg)	38 (30–45)	43 (36–49)
Creatinine (mg/dL)	0.9 (0.7–1.1)	1.4 (1.0–2.3)**
GFR (mL/min)	67 (53–81)	42 (21–64)**
NT-proBNP (ng/L)	1,510 (581–3,538)	5,278 (1,575–27,500)**
Logistic ES I (%)	15.3 (11.3–23.2)	35.8 (25.1–78.9)**
Logistic ES II (%)	3.3 (2.5–5.7)	14.4 (6.6–25.3)**

*P < 0.05, **P < 0.01. Data are presented as median, interquartile range, frequency, and percentage. AV: atrioventricular; CABG: coronary artery bypass grafting; COPD: chronic obstructive pulmonary disease; ECG: electrocardiogram; ES: EuroSCORE; GFR: glomerular filtration rate; ICD: implantable cardioverter defibrillator; LVEF: left ventricular ejection fraction; LVEDV: left ventricular end-diastolic volume; MI: myocardial infarction; NT-proBNP: N-terminal pro-brain natriuretic peptide; NYHA: New York Heart Association; PAP: pulmonary artery pressure; PCI: percutaneous coronary intervention; TIA: transient ischemic attack; TEE: transesophageal echocardiography; TTE: transthoracic echocardiography.

The time from the previous aortic valve surgery/intervention to ViV TAVI was 8.3 (4.8–10.4) years. The underlying pathology of prosthetic valve deterioration was severe regurgitation in six patients, stenosis in 10 patients, and a combination of both in nine patients. All TAVI procedures in the native AS and ViV cohorts were electively performed.

### Procedural characteristics

The procedural characteristics of the native AS and ViV cohorts are summarized in Table 3. Significant differences were found with respect to fluoroscopy time, amount of contrast dye used, frequencies of implanted 23 mm and 31 mm Allegra prostheses, performance of preimplant balloon dilatation, and the use of a cerebral protection device.

**Table 3:** Procedural Characteristics of Allegra Transcatheter Aortic Valve Implantation in Patients With Native Aortic Stenosis (AS) or Degenerated Aortic Bioprosthesis as Valve-in-Valve (ViV) Procedure

	Native AS (n = 117)	ViV (n = 25)
Intervention time (min)	60.0 (48.5–73.0)	62.0 (56.5–110.5)
Fluoroscopy time (min)	14.1 (10.3–20.0)	15.3 (13.2–32.9)*
Contrast dye (mL)	90 (65–130)	60 (40–80)**
Size of Allegra		
23 mm (n)	18 (15.4%)	16 (64.0%)**
27 mm (n)	62 (53.0%)	9 (36.0%)
31 mm (n)	35 (39.9%)	—**
Preimplanted aortic prothesis		
Epic supra (n)	—	1 (23 mm)
Hancock II (n)	—	1 (27 mm)
Magna (n)	—	1 (23 mm)
Mitroflow (n)	—	1 (23 mm), 2 (25 mm)
Mosaic (n)	—	1 (23 mm)
Perceval (n)	—	1 (size S)
Perimonut (n)	—	1 (21 mm), 2 (23 mm),3 (25 mm), 1 (27 mm)
Trifecta (n)	—	2 (23 mm)
Preimplanted TAVI prothesis		
Acurate (n)	—	1 (size M)
Evolut R (n)	—	1 (26 mm)
Sapien 3 (n)	—	1 (20 mm), 2 (23 mm),3 (26 mm)
Preimplant balloon dilatation (n)	114 (97.4%)	3 (12.0%)**
Postimplant balloon dilatation (n)	68 (58.1%)	18 (72.0%)
Concomitant planned procedures		
Diagnostic coronary angiography (n)	2 (1.7%)	1 (4.0%)
PCI (n)	5 (4.3%)	2 (8.0%)
Coronary protection(balloon/stent) (n)	2 (1.7%)	2 (8.0%)
M-TEER (n)	—	1 (4.0%)
Cerebral protection device (n)	1 (0.9%)	3 (12.0%)*
ECMO (n)	—	1 (4.0%)

*P < 0.05, **P < 0.01. Data are presented as median, interquartile range, frequency, and percentage. ECMO: extracorporeal membrane oxygenation; M-TEER: transcatheter edge-to-edge repair of the mitral valve; TAVI: transcatheter aortic valve implantation; PCI: percutaneous coronary intervention.

### Echocardiographic findings

Postinterventional echocardiography was available for 109 of the 117 patients in the native AS cohort and in 24 of the 25 patients in the ViV cohort. Echocardiographic evaluation was performed 4 (3–5) days after intervention. Postinterventional aortic regurgitation ≥ moderate was found in five of the 109 patients with native AS (4.5%) (Fig. 2), and no patient had a mean gradient of ≥ 20 mm Hg (Fig. 3). Conversely, in the ViV cohort, ≥ moderate aortic regurgitation was absent in all patients, as expected (Fig. 2); however, two patients had a mean gradient of ≥ 20 mm Hg (Fig. 3). In the native AS cohort, mean and maximal aortic pressure gradients decreased from 44 (36–54) mm Hg and 72 (60–87) mm Hg before the intervention to 6 (5–9) mm Hg and 12 (9–15) mm Hg after Allegra TAVI (each P < 0.01) (Fig. 3). In the ViV cohort, mean and maximal transvalvular pressure gradients decreased from 34 (27–43) mm Hg and 59 (44–70) mm Hg to 9 (7–11) mm Hg and 16 (14–23) mm Hg, respectively (each P < 0.01) (Fig. 3). The pressure gradients at 30 days of follow-up showed no significant difference compared to those determined at 4 days after TAVI (native AS: mean 7 (5–9) mm Hg, maximal 12 (10–15) mm Hg; ViV: mean 10 (8–13) mm Hg, maximal: 18 (14–25) mm Hg) (Fig. 3).

**Figure 2:**
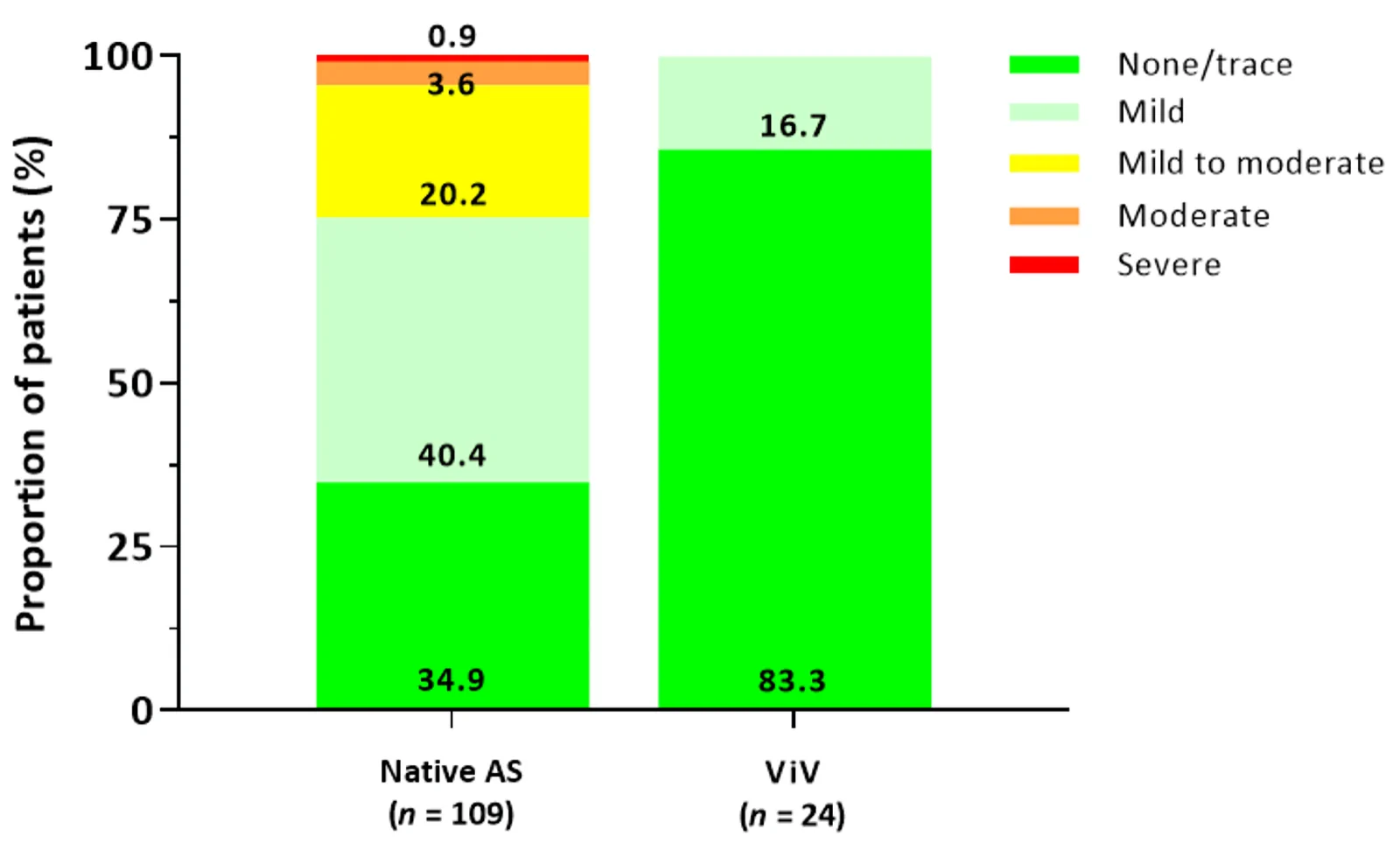
Postinterventional regurgitation after Allegra transcatheter aortic valve implantation in patients with native aortic stenosis (AS) or degenerated aortic bioprosthesis as valve-in-valve (ViV) procedure.

**Figure 3:**
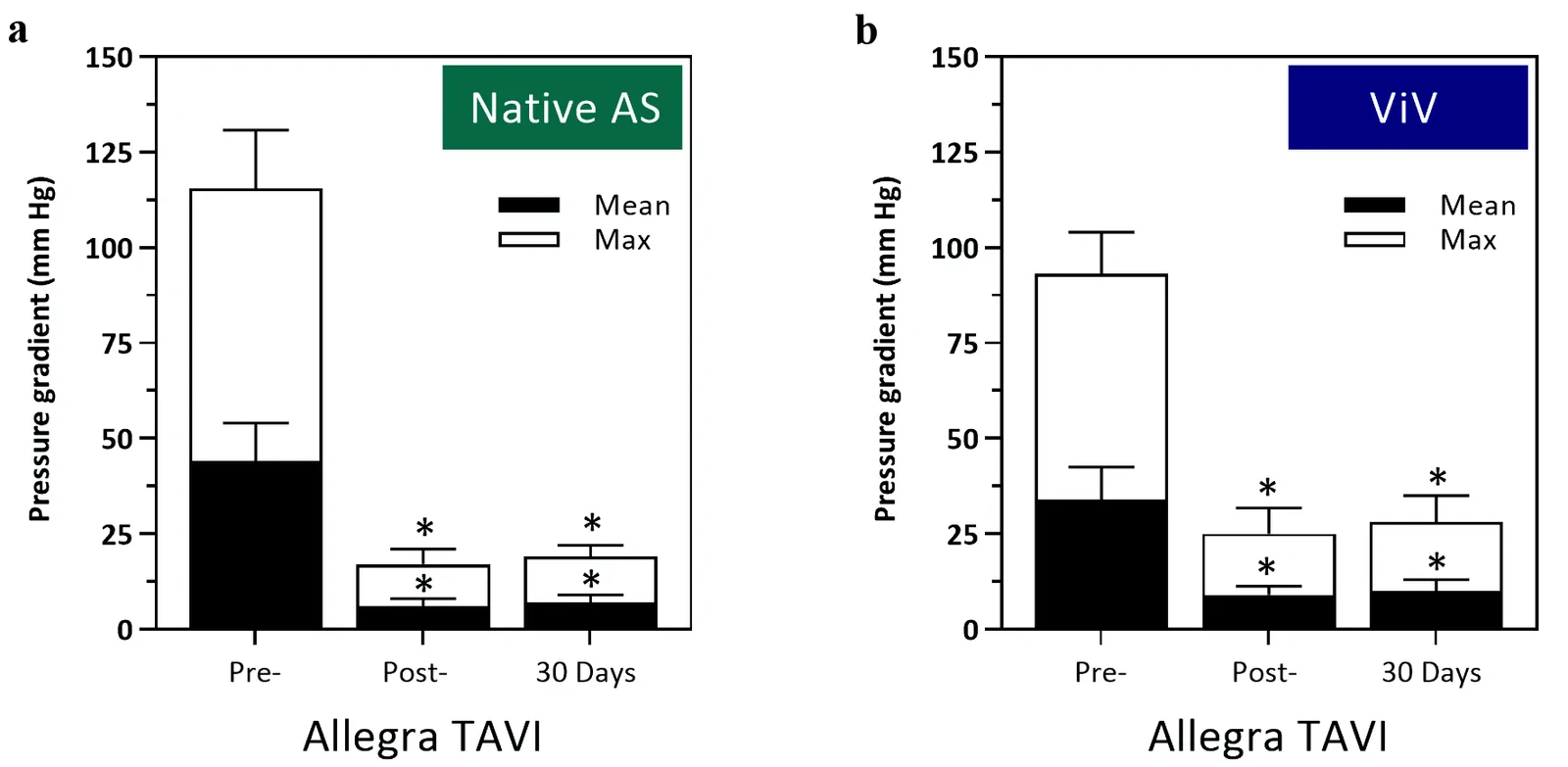
Mean and maximal (max) transvalvular pressure gradients pre-, post-, and after 30 days of Allegra transcatheter aortic valve implantation (TAVI) in patients with native aortic stenosis (AS, n = 109) or degenerated aortic bioprosthesis as valve-in-valve (ViV, n = 24) TAVI. * P < 0.01 versus pre-Allegra TAVI, each.

### Outcome

The early outcomes are summarized in Table 4. In the native AS cohort, technical success according to the VARC-3 criteria was achieved in 107 of the 117 patients (91.5%). In cases of unsuccessful Allegra implantation, one patient experienced dissection of the ascending aorta even before the Allegra prosthesis was released. This patient was converted to open-heart surgery for replacement of the aortic root and ascending aorta but did not recover and died 14 days after surgery. Another patient had intraprocedural obstruction of the right coronary artery that could not be treated interventionally (Table 4). Despite emergency coronary artery bypass surgery, the patient died 2 days later with multi-organ failure due to multiple cerebral infarctions. In two patients, the Allegra prosthesis (both 31 mm) was dislocated out of the annulus by balloon postdilatation and embolized to the ascending aorta. In these patients, the Allegra prosthesis was retracted into the descending aorta, where it was fixed with an aortic stent. TAVI was then performed using a balloon-expandable THV. However, one of the two patients with prosthesis dislocation died on the first postinterventional day due to refractory cardiogenic shock. In another patient, the Allegra prosthesis (31 mm) was initially implanted too deeply into the left ventricular outflow tract, so that embolization into the ascending aorta occurred when the prosthesis was attempted to be pulled up into the annulus. TAVI was then performed with a balloon-expandable THV, also fixing the embolized Allegra prosthesis. Five other patients experienced vascular complications that require intervention before leaving the hybrid operating room.

**Table 4:** Outcome After Allegra Transcatheter Aortic Valve Implantation in Patients With Native Aortic Stenosis (AS) or Degenerated Aortic Bioprosthesis as Valve-in-Valve (ViV) Procedure

	Native AS (n = 117)	ViV (n = 25)
Periprocedural mortality (≤ 30 days) (n)	8 (6.8%)	3 (12.0%)
Neurologic events		
Stroke/TIA (n)	3 (2.6%)	—
Delirium (n)	4 (3.4%)	3 (12.0%)
Bleeding and transfusions		
Type 1 (n)	1 (0.9%)	2 (8.0%)
Type 2 (n)	33 (28.2%)	5 (20.0%)
Type 3 (n)	3 (2.6%)	3 (12.0%)
Type 4 (n)	—	—
Vascular complications		
Major (n)	6 (5.1%)	1 (4.0%)
Minor (n)	6 (5.1%)	1 (4.0%)
Cardiac structural complications		
Major (n)	1 (0.9%)	—
Minor (n)	—	—
Other acute procedural and technical valve-related complications		
Intraprocedural conversion to open surgery (n)	2 (1.7%)	1 (4.0%)
Valve embolization (n)	3 (2.6%)	2 (8.0%)
Ectopic valve deployment (n)	3 (2.6%)	1 (4.0%)
Conduction disturbances and arrhythmias		
New bundle branch and/or AV block (n)	6 (5.1%)	—
New atrial fibrillation (n)	1 (0.9%)	—
New permanent pacemaker (n)	22 (18.8%)	—*
Acute kidney injury		
Stage 1 (n)	6 (5.1%)	—
Stage 2 (n)	—	—
Stage 3 (n)	1 (0.9%)	—
Stage 4 (n)	3 (2.6%)	—

*P < 0.05. Data are presented as frequencies and percentages. AV = atrioventricular, TIA = transient ischemic attack.

In the ViV cohort, technical success was achieved in 23 of the 25 patients (92.0%). In one case of unsuccessful Allegra (27 mm) implantation, the prosthesis was pushed out of the degenerated surgical bioprosthesis by balloon postdilatation and embolized to the left ventricle, where it was lost from the guidewire. The patient underwent open-heart surgery to retrieve the embolized THV prosthesis, and a balloon-expandable THV was implanted under visual control. After the patient was disconnected from the heart-lung machine, it was not possible to establish sufficient cardiac circulation despite all efforts. Therefore, the patient died *in tabula*. In the other patient, the Allegra prosthesis (23 mm) was pushed out of the annulus by balloon postdilatation and embolized to the ascending aorta. The Allegra prosthesis was then retracted into the distal part of the aortic arch where it was placed in a stable position. TAVI was finalized with a second Allegra THV (Table 4).

In addition to the patients who died as described above, five other patients in the native AS cohort died within the first 30 days after TAVI, resulting in a periprocedural mortality rate of 6.8% (Table 4). One patient died on day 7 after a complicated postinterventional course due to an exit block of the permanent pacemaker system. Two patients developed (aspiration) pneumonia with subsequent septic multi-organ failure and died 10 and 21 days after TAVI. Two patients died after hospital discharge at home on days 22 and 30.

In addition to the death of the patient with the Allegra prosthesis that had embolized into the left ventricle, two other patients in the ViV cohort died within the first 30 days after the procedure, resulting in a periprocedural mortality rate of 12.0% (Table 4). In detail, a multimorbid patient with renal insufficiency requiring dialysis before the intervention died 5 days after ViV TAVI following a complicated postinterventional course due to multiple organ failure. Another multimorbid patient with ischemic cardiomyopathy and severely impaired left ventricular function before the intervention died 17 days after ViV TAVI because of treatment-refractory ventricular fibrillation.

The postinterventional rate of new pacemaker implantation was significantly higher in the native AS cohort (Table 4); however, other potential complications according to the VARC-3 criteria did not differ markedly between the cohorts.

The total length of hospital stay was 10.1 (8.0–14.1) days and 12.9 (8.5–24.4) days in patients with native AS and ViV TAVI, respectively.

The median follow-up period for patients alive beyond day 30 after native AS TAVI was 2.2 (1.1–4.0) years and 1.6 (86 days–2.4) years after ViV TAVI. One-year and 3-year survival rates were 87.2% and 73.2% in the native AS cohort and 89.8% and 75.1% in the ViV cohort, respectively, and did not differ significantly (Fig. 4).

**Figure 4:**
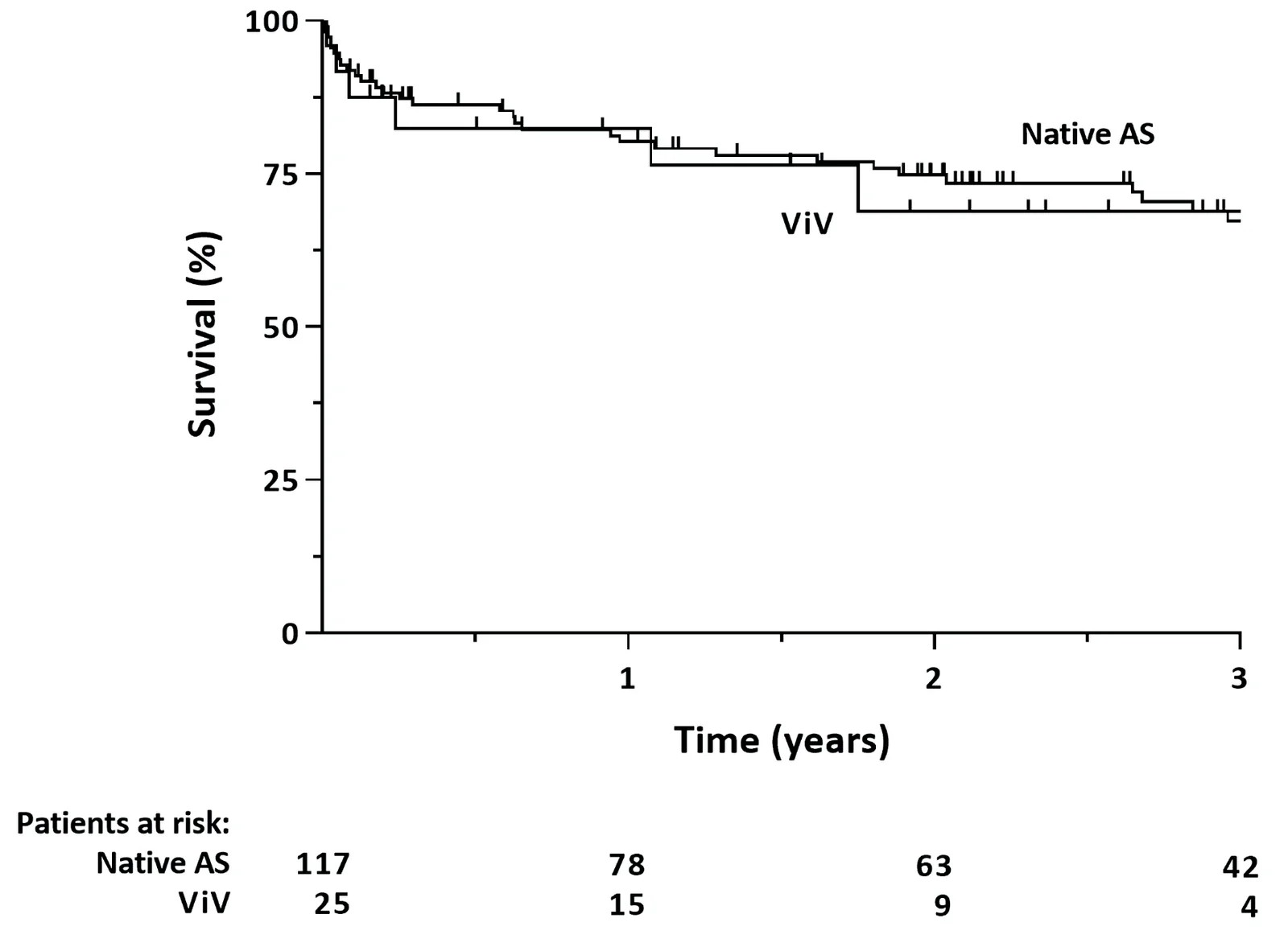
Kaplan–Meier survival rates for patients with native aortic stenose (AS) or degenerated aortic bioprosthesis (valve-in-valve (ViV)).

## Discussion

The principal findings of the present study are as follows: (1) TAVI with the Allegra prosthesis is safely feasible in patients with native AS or degenerated aortic bioprostheses and shows good hemodynamic results; (2) With regard to the possibility of prosthesis embolization, particular caution is required if an Allegra prosthesis that has not been implanted optimally needs to be re-expanded immediately after implantation.

Since patients with native AS and degenerated aortic bioprostheses are two different disease entities, and patients with redo cardiac intervention have a significantly increased risk profile (as in our patient population), the results of the two cohorts should therefore be analyzed and discussed separately.

### Allegra TAVI in patients with native AS

Our patient cohort with native AS is comparable to previously analyzed cohorts and is characterized by a higher age, predominance of women, and a risk score that fits an intermediate risk profile. As already shown in earlier studies of the Allegra THV [2, 3, 10, 11], actual postinterventional echocardiography indicated excellent pressure gradients of the prosthesis, and neither of the patients had a mean gradient ≥ 20 mm Hg. Less than moderate aortic regurgitation was achieved in 95.5% of our patients with native AS, which is within a good range compared to 84% of patients from previous Allegra studies and 72–79% of patients from other self-expandable THV studies [3, 12, 13]. Our present short-term composite endpoints after Allegra TAVI are also in line with those previously reported [2–4], although it must be remembered that previous studies were conducted almost exclusively according to the VARC-2 criteria and not, as in this study, according to the current VARC-3 criteria. Technical success was achieved in 107 of the 117 patients with native AS (91.5%). While recognizing the limitations of indirect comparisons, these results are comparable to other real-world data from self-expandable TAVI systems such as the Medtronic Evolut PRO (90%), Evolut R (89%), Evolut FX (89%) or Abbott Navitor system (94.2%) and the latest-generation balloon-expandable Edwards Sapien 3 Ultra Resilia system (92.2%) [14–16]. In cases of technical failure, half of the patients had vascular complications, and the other half had structural complications (dissection of the ascending aorta, obstruction of the right coronary artery) or prosthesis embolization. The periprocedural mortality rate (≤ 30 days) was 6.8%, which slightly but not significantly exceeds the previous 30 days mortality rates after Allegra TAVI (2.9–4.8%) [4, 11]. To date, only one working group has analyzed the long-term survival of a cohort of 103 patients after Allegra TAVI (including three patients with negligible ViV TAVI), showing a survival rate of 68.6% after three years [11]. Our long-term results (73.2% survival at three years) are almost identical to those mentioned above and, as previously stated, are consistent with data from other THV systems [11].

### Allegra ViV TAVI in patients with degenerated aortic bioprostheses

Despite increasing experience with THV and the significant technical advancement of current catheter systems, ViV procedures are still high-risk interventions. This is also reflected in the high-risk scores of these patient collectives (as in this study). As expected, no significant postinterventional periprosthetic regurgitation was actual observed in any patient with Allegra ViV TAVI; however, two patients, one with a previously implanted 20-mm Sapien THV inside a Direct Flow Medical valve and the other with a 23-mm Mosaic surgical heart valve, had a mean gradient ≥ 20 mm Hg after ViV TAVI. Nevertheless, only recently, it was found that the lowest mean pressure gradients, as well as the largest decrease in mean pressure gradients from baseline to discharge after VIV TAVI, were achieved with the Allegra THV compared to the CoreValve/Evolut R or Sapien THV in patients with previously implanted small aortic bioprostheses [6]. Technical success was achieved in 92.0% of our ViV Allegra cases, which is slightly better than the latest results from a UK multicenter registry, which reported technical success in 86.8% of Allegra ViV TAVI procedures [17].

Five valve embolizations occurred immediately after postdilatation of a suboptimally implanted Allegra valve in our series. The Allegra prosthesis is implanted by using a unique three-step release mechanism. After the initial opening of the middle portion of the crimped valve and positioning of the marker band in line with the annulus (step 1), the inflow is released by pushing the “tip release” (step 2). This step is carried out under “rapid pacing” and, together with the third step, which means retraction of the nose cone and release of the top of the valve stent, represents the most important parts of the positioning process, as unpredictable changes in the alignment of the prosthesis can occur at this point. Although valve embolization has also been reported in previous Allegra studies [4, 11], our data once again underline the need for caution when re-expanding an Allegra valve with a balloon that had not been optimally implanted immediately before. Furthermore, the next generation of the Allegra THV system will feature modifications to the delivery system that address these implantation issues, ensuring even greater stability of the valve during the release process [7].

### Limitations

Our findings need to be seen in light of several limitations, such as the lack of a THV comparison group. However, unlike the patients described here with degenerated stented bioprostheses, most of our patients with degenerated bioprostheses are those who previously received a stentless Medtronic Freestyle prosthesis. In these patients, we use an Edwards Sapien prosthesis in the event of a ViV procedure, as we have previously published [18, 19]. Another system used in our center is the Abbott Navitor TAVI system; however, we only use this for patients with native aortic valve stenosis and not for ViV procedures.

Further limitations include the fact that the study was conducted only at a single center, as well as the relatively small number of patients in both cohorts. Therefore, larger pooled studies are needed to assess safety and efficacy in both short-term and long-term follow-up.

The results of large cohorts such as the FOLLOW registry (Clinical outcomes of the NVT Allegra TAVI system TF in failing calcified aortic heart valves in a real-world patient population with elevated surgical risk; NCT03613246) will certainly provide more data on this, especially regarding the long-term course of patients.

### Conclusions

TAVI using the self-expanding Allegra THV system is safe, leads to good functional results, and may offer an effective alternative to the more established THV for the treatment of patients with native AS or those with failed surgical aortic bioprostheses.

## Data Availability

All data can be obtained on request from the corresponding author.
